# Exposure to Diflubenzuron Results in an Up-Regulation of a Chitin Synthase 1 Gene in Citrus Red Mite, *Panonychus citri* (Acari: Tetranychidae)

**DOI:** 10.3390/ijms15033711

**Published:** 2014-02-28

**Authors:** Wen-Kai Xia, Tian-Bo Ding, Jin-Zhi Niu, Chong-Yu Liao, Rui Zhong, Wen-Jia Yang, Bin Liu, Wei Dou, Jin-Jun Wang

**Affiliations:** Key Laboratory of Entomology and Pest Control Engineering, College of Plant Protection, Southwest University, Chongqing 400716, China; E-Mails: xiawenkai0409@126.com (W.-K.X.); dingdingsky315@126.com (T.-B.D.); jinzhiniu@yahoo.com (J.-Z.N.); leochongyu@163.com (C.-Y.L.); zhongrui19890326@gmail.com (R.Z.); yangwenjia10@126.com (W.-J.Y.); lexylexylexy@163.com (B.L.); anwdou@gmail.com (W.D.)

**Keywords:** *Panonychus citri*, chitin synthase 1, diflubenzuron, insect growth regulators, pest control

## Abstract

Chitin synthase synthesizes chitin, which is critical for the arthropod exoskeleton. In this study, we cloned the cDNA sequences of a chitin synthase 1 gene, *PcCHS1*, in the citrus red mite, *Panonychus citri* (McGregor), which is one of the most economically important pests of citrus worldwide. The full-length cDNA of *PcCHS1* contains an open reading frame of 4605 bp of nucleotides, which encodes a protein of 1535 amino acid residues with a predicted molecular mass of 175.0 kDa. A phylogenetic analysis showed that *PcCHS1* was most closely related to *CHS1* from *Tetranychus urticae*. During *P. citri* development, *PcCHS1* was constantly expressed in all stages but highly expressed in the egg stage (114.8-fold higher than in the adult). When larvae were exposed to diflubenzuron (DFB) for 6 h, the mite had a significantly high mortality rate, and the mRNA expression levels of *PcCHS1* were significantly enhanced. These results indicate a promising use of DFB to control *P. citri*, by possibly acting as an inhibitor in chitin synthesis as indicated by the up-regulation of *PcCHS1* after exposure to DFB.

## 1. Introduction

Chitin, a homopolymer of β-1,4-linked *N*-acetylglucosamines, is the second most abundant natural polymers after cellulose. It is widely distributed in arthropods, nematodes, and fungi [1,2]. In arthropods, it is the principal component of the cuticle and the peritrophic membrane (PM) which lines the midgut epithelium [3]. During growth and development, part of the old cuticle is digested, while new chitin is deposited and synthesized. The crucial step of the chitin biosynthetic pathway in arthropods is associated with chitin synthase (*CHS*). CHSs are large transmembrane proteins with slightly acidic isoelectric points and theoretical molecular masses ranging from 160 to 180 kDa, which belong to two families of glycosyltransferases that catalyze transfers of sugar moieties from activated sugar donors to specific acceptors in a glycosidic bond [4,5].

The first cDNA sequence encoding an insect or mite chitin synthase gene was obtained from *Lucilia cuprina* [6]. Since then, several sequences of insect or mite chitin synthases have been isolated and sequenced in many species, including *Aedes aegypti* [7], *Anopheles gambiae* [8], *Anopheles quadrimaculatus* [9], *Bactrocera dorsalis* [10], *Choristoneura fumiferana* [11], *Drosophila melanogaster* [12], *Ectropis oblique* [13], *Locusta migratoria manilensis* [14], *Manduca sexta* [15], *Ostrina furnacalis* [16], *Plutella xylostella* [17], *Spodoptera exigua* [18], *S. frugiperda* [19], *Tribolium castaneum* [2], and *Tetranychus urticae* [20]. Generally, most insects or mites possess two types of chitin synthase genes, namely *CHS1* and *CHS2* (also referred to as CHS-A and CHS-B) [19,21]. During growth and development, *CHS1* is mainly responsible for forming the chitin used in cuticles and the cuticular linings of the foregut, hindgut and trachea. *CHS2*, however, is specifically produced by midgut epithelial cells and is associated with the PM [1,22]. Presently, in hemipteran insects, such as *Nilaparvata lugens* and *Aphis glycines*, only *CHS1* has been identified. During the course of evolution, hemipteran insects seem to have lost the PM but evolved an extracellular lipoprotein membrane, the perimicrovillar membrane, surrounding the midgut microvilli cells [23–25]. Since they lack the PM, these insects may have also lost the *CHS2* gene during evolution [23,24].

The citrus red mite, *Panonychus citri* (McGregor), has a worldwide distribution and is an economically important citrus pest that rapidly adapts to hosts and has evolved resistance to acaricides [26–29]. Although biological control has been successful in mite management, chemical control is still the main strategy against this pest in the field. According to the Arthropod Pesticide Resistance Database [30], *P. citri* has developed 51 cases of resistance involving 27 acaricides, including amitraz, azocyclotin, abamectin, dicofol, dimethoate, hexythiazox, pyridaben, parathion, spirotetramat, and tetradifon. Therefore, it is urgent to find new pesticides or new targets with serious resistance to replace the acaricides or to develop new acaricides. Due to the fact that the chitin synthesis pathway is absent in vertebrates and plants, chitin synthesis inhibitors are safe for humans and are also promising for controlling mites [5]. Diflubenzuron (DFB), the main representative of benzoylphenylureas which were discovered in the 1970s, is an insect growth regulator in chitin synthesis [31,32]. DFB treatments of *A. quadrimaculatus* [9], *A. glycines* [8], and *T. urticae* [20] increased *CHS1* mRNA levels in these species, but no effects were reported in *T. castaneum* [33] and *Pieris brassicae* [34]. However, the precise mode of action of DFB remains elusive. In *T. castaneum*, genomic tiling array of 11,000 genes found only about 6% of genes showed differential expression in response to DFB treatment. At the same time, genes involved in chitin metabolism were unaffected by treatment by DFB [33]. Nevertheless, in *T. urticae*, a target-site resistance mutation raised the possibility that a chitin biosynthesis inhibitor, such as DFB, may act to inhibit chitin synthases [20]. However, the *CHS* profile in *P. citri* and its interaction with DFB remains unclear. In this study, we report (1) a full-length cDNA encoding chitin synthase 1 (*PcCHS1*) from *P. citri*; (2) the expression profiles of *PcCHS1* at various developmental stages; and (3) the effects of DFB on the expression of *PcCHS1* during the larval stage of *P. citri*.

## 2. Results

### 2.1. Analyses of the cDNA and Protein Sequence of PcCHS1

**Figure 1.**
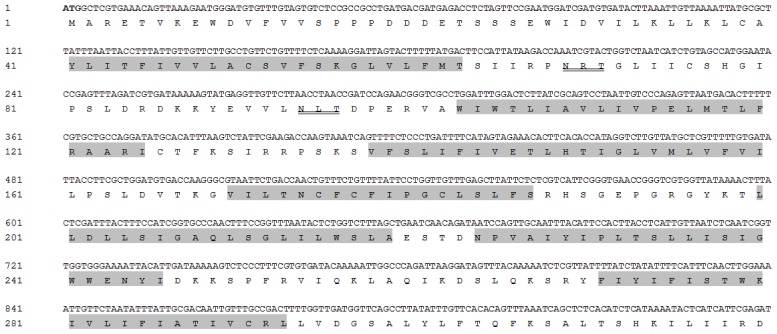

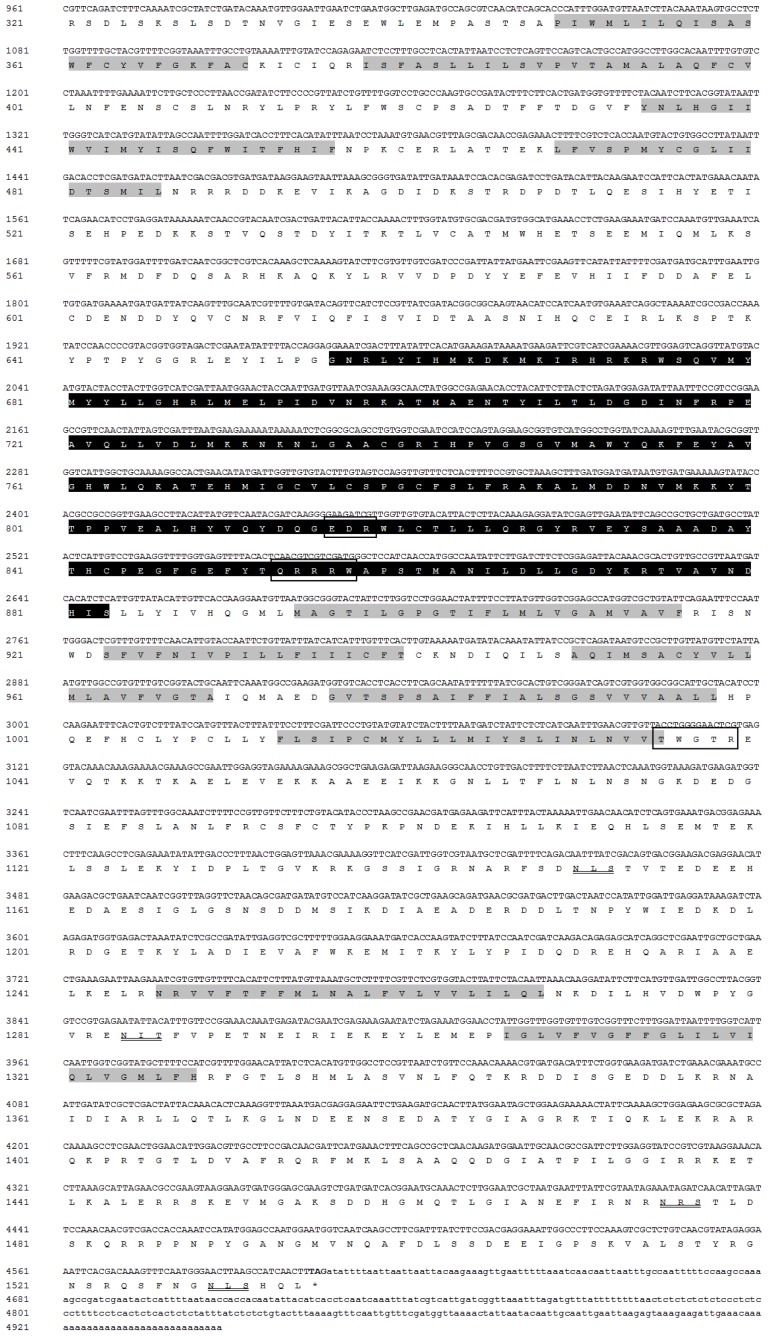
Nucleotide and deduced amino acid sequences of the chitin synthase 1 gene, *PcCHS1*, from the citrus red mite, *Panonychus citri* (Acari: Tetranychidae), cDNA (KF241748). The start codon (ATG) is indicated in black and the stop codon (TGA) in black with an asterisk. The chitin synthase signature motifs (EDR and QRRRW) are boxed with a black background, while TWGTR is boxed. Eighteen putative transmembrane regions are shaded. The six *N*-glycosylation sites are indicated with double lines. The putative catalytic domain is in white with a black background.

The complete cDNA sequence of *PcCHS1* (KF241748) contains an open reading frame (ORF) of 4605 bp encoding 1535 amino acid residues with a predicted molecular mass of 175.0 kDa and an isoelectric point of 6.41 (Figure 1). *PcCHS1* was predicted to have 18 transmembrane helices and three domains including: domain A (*N*-terminal domain) with 11 transmembrane helices; domain B (a high conserved catalytic domain) with two signature motifs, EDR (816–818) and QRRRW (853–857); and domain C (*C*-terminal domain) with seven transmembrane helices and a signature motif TWGTR (1035–1039), which was assumed to play a crucial role in chitin translocation. In addition, six potential *N*-glycosylation sites at positions 69, 94, 1150, 1284, 1475, and 1529 were predicted by employing NetNGLyc 1.0 software (Technical University of Denmark, Copenhagen, Denmark).

**Figure 2.**
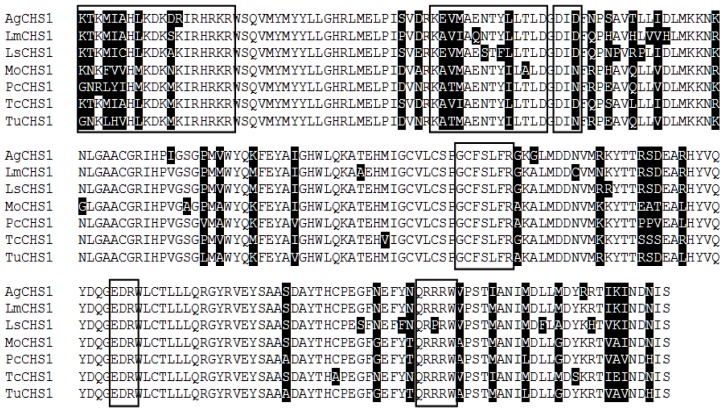
ClustalW alignment of putative conserved catalytic domains of chitin synthases genes from three mites and four insect species. Conserved domains with identical amino acid residues are shown with white backgrounds. Six black boxes of amino acid residues refer to the highly conserved regions in glycosyltransferases enzymes [19]. The chitin synthase sequences used in the alignment are: *AgCHS1: Anopheles gambiae*, *LmCHS1: Locusta migratoria manilensis; LsCHS1: Laodelphax striatella; MoCHS1: Metaseiulus occidentalis; PcCHS1: Panonychus citri; TcCHS1: Tribolium castaneum; TuCHS1: Tetranychus urticae*.

Multiple protein alignments of the catalytic domains of chitin synthases from Arachnida and insects showed highly conserved identity levels of 96.1% in *T. urticae* (tetur03g08510) and 90.4% in *M. occidentalis* (XP003741992), as well as 82.0%, 81.6%, 81.1%, and 79.4% similarity in *T. castaneum* (AY291476), *A. gambiae* (XP_321336.4), *L. migratoria manilensis* (GU067731), and *L. striatella* (JQ040011), respectively (Figure 2).

### 2.2. Phylogenetic Analysis of PcCHS1

**Figure 3.**
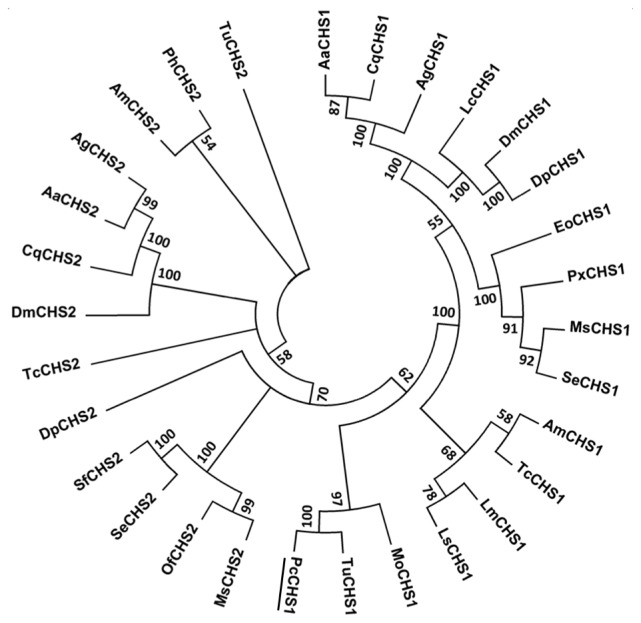
Phylogenetic analysis between the chitin synthase 1 gene, *PcCHS1*, from the citrus red mite, *Panonychus citri* (Acari: Tetranychidae), and the known acari and insect chitin synthase genes. The phylogenetic tree was constructed by MEGA 5.04 based on the neighbor-joining method according to amino acid sequences. Bootstrap support values with 1000 are shown on the branches. The chitin synthase sequences used to generate the tree are listed in Table 1.

As *P. citri* and *T. urticae* are from the same family, Tetranychidae, the alignment of amino acid sequences was constructed among *CHSs* genes in this family and other insect species using MEGA5.04. A phylogenetic analysis showed that *CHS1* and *CHS2* were located in different phylogenetic groups. Additionally, the *CHS1* gene from *T. urticae* and *P. citri* clustered into the *CHS1* family and seemed to share a single clade. A high bootstrap value of 1000 confirmed the phylogenic tree (Figure 3). This result indicated that similar physiological functions and evolutionary relatedness may exist between the *CHS1* in *T. urticae* and *P. citri*.

### 2.3. Development-Specific Expression Patterns of PcCHS1

**Figure 4.**
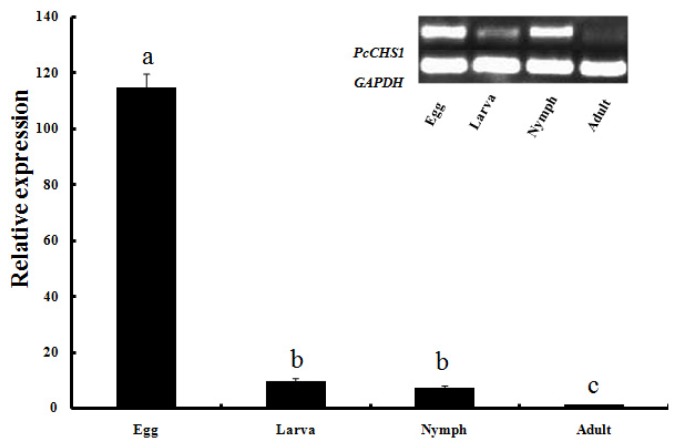
Expression levels of the chitin synthase 1 gene, *PcCHS1*, across different developmental stages of citrus red mite, *Panonychus citri* (Acari: Tetranychidae). Relative transcript levels of *PcCHS1* in egg, larva, nymph, and adult stages were examined using qPCR. The relative expression was calculated based on the value of the lowest expression level, which was assigned the arbitrary value of 1. Data are means (±SE) of three biological replications per developmental stage. Different letters on the error bars show significant differences by an ANOVA test (*p* < 0.05). *GAPDH* was used as the reference gene.

To understand the function of *PcCHS1*, expression levels of *PcCHS1* in different developmental stages of *P. citri* development, including egg, larva, nymph and adult were evaluated by qPCR (Figure 4). The results showed that *PcCHS1* mRNA was expressed in all stages, suggesting that *PcCHS1* has roles in biological processes throughout all developmental and growth stages. More specifically, *PcCHS1* was highly expressed at the eggs stage, whereas it was the lowest in the adult stage. The relative expression levels of *PcCHS1* in eggs, larvae, and nymphs were 114.8-, 9.6-, and 7.3-fold significantly higher than the level in adults (*p <* 0.05).

### 2.4. Effects of DFB Treatment on the Expression of PcCHS1

**Figure 5.**
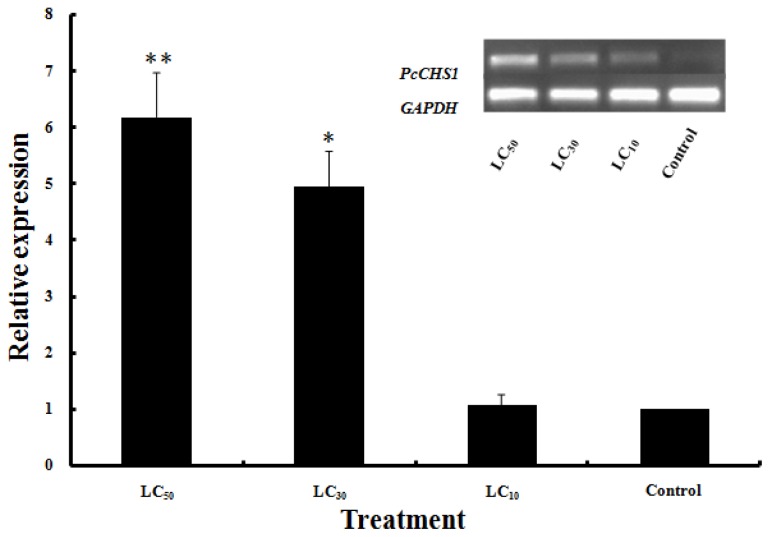
Effects of diflubenzuron (DFB) treatment on the expression pattern of the chitin synthase 1 gene, *PcCHS1* from the citrus red mite, *Panonychus citri* (Acari: Tetranychidae). Relative expression of the *PcCHS1* of *P. citri* exposed to 0.17, 1.31 and 5.32 mg/L DFB (LC_10_, LC_30_ and LC_50_) in 0.1% Triton-100 at the larval stage for 6 h using a leaf-dip bioassay were analyzed using qPCR (*n* = 3). Water, containing 0.1% Triton-100, treated *P. citri* were used as the controls. The error bars were generated after examining the relative level of *PcCHS1* in *P. citri*. An asterisk (*) on the error bar indicates a significant difference between DFB treatment and control; * (*p* < 0.05) or ** (*p* < 0.01, *t*-test). *GAPDH* was used as a reference gene.

Exposure of *P. citri* larvae to DFB caused the obvious phenomena of abortive molting and high mortality. However, the larvae in the control group molted normally and developed into nymphs after 1 d. After treatment with DFB at low-lethal concentration (LC_10_), sub-lethal concentration (LC_30_) and median lethal concentration (LC_50_), the relative expression level of *PcCHS1* was 1.1-, 5.0- and 6.2-fold higher than in the control. A statistical analysis suggested that *PcCHS1* was up-regulated significantly with the LC_30_ and LC_50_ doses of DFB after 6 h but had no significant difference after the LC_10_ dose of DFB when compared with the control. There was no significant difference in the expression patterns between the LC_30_ and LC_50_ DFB doses (Figure 5).

## 3. Discussion

Chitin synthases play key roles in chitin biosynthesis during growth and development, and have been studied in fungal, nematode and in various insect species. Until now, at least 15 species of insect chitin synthases have been cloned and characterized [23], while studies on mite chitin synthases are still relatively scan except *T. urticae* [20]. Insect chitin synthases have been classified into two different groups, *CHS1* and *CHS2* [35]. *CHS1* is primarily expressed in cuticle and tracheal cells whereas *CHS2* is exclusively expressed in the midgut epithelial cells [21,36]. To better understand the biological functions of *CHSs*, analyses of these genes in different species have received much attention. In this study, we first cloned and characterized a full-length cDNA encoding chitin synthase 1, *PcCHS1*, from *P. citri*. The *PcCHS1* cDNA contains three domains with all the signature motifs, including EDR and QRRRW in the highly conserved catalytic domain, which faces the cytoplasmic side, and TWGTR, which faces the extracellular side (Figure 1).These motifs are considered as chitin synthase expression sequence tags. The alignment and phylogenic analyses showed that the *CHS* from *P. citri* belonged to the *CHS1* group.

Alternative splicing expands the diversity of mRNA transcripts and augments modulating gene functions [37]. Research on insect *CHS* genes revealed that only the *CHS1* genes have two alternative exons that produce two splicing variants, *CHS1a* and *CHS1b*, having a short 177 bp region of difference encoding 59 amino acids in the second to the last predicted transmembrane helix [14,21,38,39]. These two alternative exons located in the middle ORF region have been reported in *T. castaneum* [2], *P. xylostella* [17], *M. sexta* [15], *S. exigua* [18], *O. furnacalis* [16] and *L. migratoria manilensis* [14]. Additionally, a novel alternative site was found in *O. furnacalis* (*OfCHSA-2a* and *OfCHSA-2b*) and *B. mori* using gDNA sequence alignments [40]. To investigate the splice variants of *P. citri CHS1*, a pair of special primers [10,14,17,23] designed based on *T. castaneum* splice variants region were synthesized to amplify cDNA using templates from different developmental stages. However, our efforts to discriminate *P. citri CHS1* splice variants in any developmental stage failed. Genome fragments amplification of chitin synthase in the *P. citri* genome also revealed no splice variants exist. It seems that *P. citri* have no alternate exon-a, only exon-b, which is similar to the results from aphids [24]. It is interesting that this occurs only in these two species. Whether the function of exon-a has been successfully replaced or exon-a is unnecessary in chitin biosynthesis is unknown and needs to be explored in depth. Moreover, the relationship between alternative splicing and evolution still requires further investigation.

The expression of *PcCHS1* was detected in all four tested developmental stages of *P. citri*. The expression levels in the eggs were significantly higher than in the adults, which is similar to the levels in *A. glycines*. In *A. glycines*, *AyCHS* has an exceedingly high transcription level in aphid embryos. This high level of *AyCHS* might reflect its unique role during embryonic development. Embryonic molting can form a large amount of chitin, which leads to the higher expression of *CHS1* [24]. However, in *L. migratoria manilensis*, expression of *LmCHS1* reached its lowest level in eggs and highest in adults [14]. While in *B. dorsalis*, *CHS1* was mainly expressed during larva-pupal and pupal-adult transitions, and the highest level was in day-1 adults [10]. In *O. furnacalis*, *OfCHS-2a* expression was mainly present in newly laid eggs and larval-larval molting, as well as larval-pupal transformation, whereas *OfCHS-2b* was mainly expressed during larval-pupal molting [16]. Our results of high expression levels in *P. citri* eggs might reflect the special expression during egg molting and embryo development in this mite.

DFB is an insect growth regulator that has been successfully established as a potent insecticide for pests control in forestry and agriculture. It can selectively inhibit chitin biosynthesis in insects but not in fungi [41]. When exposed to this chitin synthesis inhibitor, insects may develop disturbed cuticle formations, abnormal depositions of procuticles and abortive molting [9,42]. Early studies using radiolabeled precursors suggested that DFB inhibited chitin synthesis because the growth of the chitin polymer was impaired [43,44]. When treated with DFB, the cuticles of larvae might fail to withstand the increased turgor and be unable to provide sufficient support to fight against harsh environments. Larvae may be unable to digest old cuticles or they may synthesize delicate and malformed cuticles resulting in mass mortality [9]. Since the chitin biosynthetic pathway is absent in humans and vertebrates, DFB have the potential to be used in IPM strategies for pest control and *P. citri* in citrus fields. In this study, the significant up-regulation of *PcCHS1* in *P. citri* was detected at 6 h in larvae after treatment with DFB. We have shown that under laboratory conditions, sub-lethal concentrations and median lethal concentrations of DFB as treatments significantly increased the expression of *PcCHS1* in *P. citri* larvae (Figure 5). Similarly, about 2-fold up-regulation of *CHS1* in *A. quadrimaculatus* [9] and *A. glycines* [24] exposed to DFB has been reported. Because *CHS1* is only expressed in the epidermis, it may be primarily responsible for the synthesis of chitin. The above-mentioned results indicate that exposure to DFB can reduced the chitin content due to the inhibition of chitin synthase activity or, increased *CHS1* expression may indicate the existence of a feedback regulatory mechanism that compensates for the enzymes content [9]. However, previous studies revealed that DFB had no effect on *CHS1* expression in *T. castaneum* [21] and *D. melanogaster* [45], suggesting that DFB may promote the separation and digestion of the cuticle during molting. Since chitin biosynthesis is a very intricate process, the influence of DFB on the insect chitin synthesis mechanism remains to be explored in depth. Future work should pay attention to the biological significance of *PcCHS1* expression and the relevant molecular mechanism of DFB action in *P. citri*.

*P. citri* is a globally polyphagous pest that devastates both green fruit trees and deciduous trees, such as citrus, peach and pear [46]. In recent years, *P. citri* outbreaks have frequently occurred in southern China. Many types of insecticides have been heavily applied to control the pest, inevitably leading to serious resistance [30]. Recently, chitin synthase has been used very successfully as a target gene for RNA interference for *T. castaneum*, *B. dorsalis*, *O. furnacalis* and *T. urticae* control [10,16,21,47–49]. In addition, the silencing of insect chitin synthesis by disrupting its function may be an effective method for future control strategies by targeting *PcCHS1* in *P. citri*. Further, this approach could offer a novel *P. citri* management policy in the field.

## 4. Experimental Section

### 4.1. *P. Citri,* Leaf-dip Bioassay

The *P. citri* used in this study were obtained from a local citrus orchard at the Citrus Research Institute, Chinese Academy of Agricultural Sciences, Chongqing (29°45′N 106°22′E), China, in 2013. The mites were reared on fresh citrus seedlings without exposure to pesticides in an insect-rearing room at 25 ± 1 °C, 75%–80% relative humidity, with a 14:10 h light:dark photoperiod. To obtain enough individuals of different developmental stages for the experiments, more than 500 leaf discs were prepared. Flash leaves were gathered randomly from *Camellia reticulate* Blanco trees from the orchards. The fresh leaves, without prior pesticide exposure, were washed thoroughly. Leaf discs of 3-cm diameter were placed on a 4-mm layer of water-saturated sponge in Petri dishes (9 cm diameter) [50]. Approximate 30 adult females were transferred to each leaf disc and allowed to lay eggs for 12 h before being removed. After the uniform eggs hatched, the offspring were kept until the progeny had developed into 3- to 5-d old females [51].

For the leaf-dip bioassay, DFB (LKT Laboratories Inc., Saint Paul, MN, USA) was used to treat the larval mites, and 0.1% Triton-100 (Beijing Dingguo Changsheng Biotech. Co. Ltd., Beijing, China) was used as the nonionic surfactant [51]. The LC_10_, LC_30_ and LC_50_ of DFB were defined as the treatment concentrations. First, 150 mg of DFB was dissolved in 30 mL of acetone, producing a 5000 mg/L stock solution. Then pipette 3.5, 26.2, and 106.3 μL stock solution to 100 mL distilled water diluted to 0.17, 1.31 and 5.32 mg/L corresponding to LC_10_, LC_30_ and LC_50_ working solutions, respectively. Each detached citrus leave retaining 30 larval mites were dipped for 5 s in the test solution containing 0.1% Triton-100. Leaves treated with distilled water containing only 0.1% Triton-100 were used as a control. When the leaves had dried, they were returned to the conditions as above. After 24 h, the surviving mites were counted and stored at −80 °C for RNA extraction.

### 4.2. RNA Isolation and Reverse Transcription

Total RNA used for cloning cDNA and analyzing *PcCHS1* profiles was isolated using the RNeasy^®^ Plus Micro Kit (Qiagen, Hilden, Germany), and a gDNA elimination column was applied to remove genomic DNA according to the manufacturer’s instructions. The cDNA was synthesized using the total RNA and rapid amplification of cDNA ends (RACE). Total RNA was dissolved in 20 μL H_2_O treated with DEPC and stored at −80 °C for future use. For the total RNAs, quantities were assessed at 260 nm and the purities evaluated at an absorbance ratio of OD_260/280_ using a Nanovue UV-Vis spectrophotometer (GE Healthcare, Fairfield, CT, USA). RNA integrity was affirmed by 1% agarose gel electrophoresis. The first-strand cDNA was prepared with 500 ng of RNA in a 10 μL reaction mixture using PrimeScript^®^ 1st strand cDNA synthesis Kit (TaKaRa, Dalian, China) and oligo (dT)_18_ primers and stored at −20 °C.

### 4.3. Cloning and Sequencing of PcCHS1

**Figure 6.**
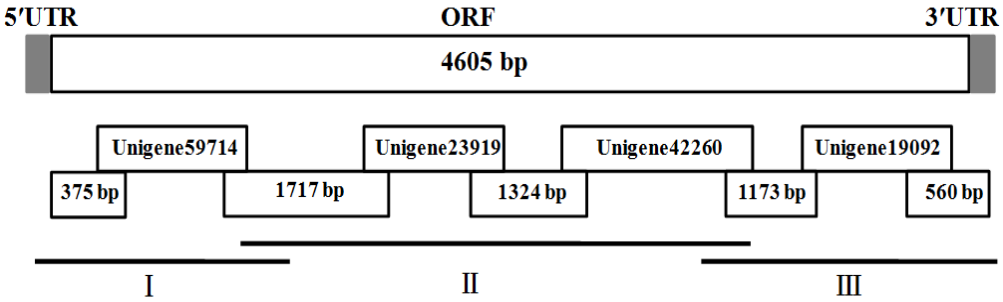
Schematic diagram of the strategy to amplify cDNA of the chitin synthase 1 gene, *PcCHS1*, from the citrus red mite, *Panonychus citri* (Acari: Tetranychidae).The upper bar represents of the cDNA sequence in *P. citri*. Four PCR fragments were obtained from the *P. citri’*s transcriptome data. Fragment II was amplified using specific primers. Fragments I and III were obtained by 5′- and 3′-RACE, respectively.

Four *CHS1* fragments were obtained based on the results of *P. citri* high-throughput transcriptome sequencing as per our previous work [52]. The specific primers and cloning strategy were designed to obtain the full-length *CHS1* (Table 2 and Figure 6). Specific PCR and nested PCR reaction were performed in a C1000™ Thermal Cycler (BIO-RAD, Hercules, CA, USA). The 5′- and 3′-RACE ends were amplified using SMARTer™ RACE cDNA amplification according to the instructions (Clontech, Palo Alto, CA, USA). The total volume of PCR was 25 μL with 2.5 μL 10× PCR buffer (Mg^2+^ free), 2.0 μL dNTPs (2.5 mM), 2.0 μL Mg^2+^ (2.5 mM), 1 μL cDNA templates, 1 μL each primer (10 mM), 0.25 μL rTaq™ polymerase (TaKaRa) and 15.5 μL ddH_2_O. The PCR program was performed by an initial denaturation for 3 min at 94 °C, followed by 34 cycles of 94 °C for 30 s, 55 to 60 °C (based on the primer annealing temperatures) for 30 s, 72 °C extension for 1 to 2 min and final extension for 10 min at 72 °C. The amplified PCR fragments were gel-purified with the Gel Extraction Mini Kit (Watson Biotechnologies, Shanghai, China) and then ligated into pGEM^®^-Teasy vector (Promega, Fitchburg, MA, USA). Recombinant plasmids were sequenced using an ABI Model 3100 automated sequencer (Invitrogen Life Technologies, Shanghai, China).

### 4.4. Sequence Retrieval and Phylogenetic Analysis

The sequence analysis for conserved domains was performed using the NCBI BLAST website (http://www.ncbi.nlm.nih.gov/Blast.cgi). The ClustalW program was used to align the deduced amino acid sequence of *CHS1* [53,54]. The molecular weight and isoelectric points of the deduced protein sequences were calculated by the ExPASy Proteomics Server (http://cn.expasy.org/tools/pi_tool.html) [55]. The signal peptide was predicted using Signa1P 3.0 (http://www.cbs.dtu.dk/service/SignalP/) [56], and the transmembrane helices were analyzed using TMHMM v.2.0 (http://www.cbs.dtu.dk/services/TMHMM-2.0/) [57]. The *N*-glycosylation sites were predicted by the NetNGlyc 1.0 Server (http://www.cbs.dtu.dk/services/NetNGlyc/) [58]. DNAMAN 6.0 (DNAMAN 6.0, Lynnon BioSoft, Quebec, Canada) was used to edit the nucleotide sequences of *PcCHS1* and the phylogenetic analysis was conducted with MEGA5.04 [59] using the neighbor-joining method to infer the evolutionary history. Bootstrap values were calculated based on 1000 replications.

### 4.5. Expression Patterns of PcCHS1 in Various *P. citri* Developmental Stages

To investigate the development-specific expression patterns of *PcCHS1*, cDNAs were prepared from four developmental stages (2000 eggs, 1500 larvae, 1000 nymphs, and 800 adults) of the mite. *PcCHS1* specific primers used for qPCR analysis were designed by primer 3.0 (http://frodo.wi.mit.edu/) [60] (Table 2). *GAPDH* (HM582445) was used as a stable reference gene across the different developmental stages [61]. The qPCR was Performed on a Mx3000P thermal cycler (Agilent Technologies, Inc., Wilmington, NC, USA) with 20 μL reaction mixtures containing 1 μL cDNA, 10 μL iQ™ SYBR^®^ Green Supermix (BIO-RAD, Hercules, CA, USA), 1 μL of each gene-specific primer (0.2 mM) and 7 μL ddH_2_O. The optimized qPCR protocol used for amplification was: 95 °C for 2 min, then 40 cycles of denaturation at 95 °C for 15 s, 60 °C for 30 s and elongation at 72 °C for 30 s. Finally, melt curve analyses (from 60 to 95 °C) were included at the end to ensure the consistency of the amplified products. A total of three biological and two technical replicates were performed for each experiment. The quantification of expression level was analyzed using the 2^−ΔΔ^*^C^*^t^ method [62].

### 4.6. Expression of PcCHS1 after DFB Exposure

To examine the effect of DFB exposure on *P. citri CHS1* expression, DFB was used to treat larval mites while using 0.1% Triton-100 as the surfactant. The LC_10_, LC_30_ and LC_50_ of DFB corresponded to 0.17, 1.31 and 5.32 mg/L, respectively, as in the leaf bioassay. After a 6 h interval, only surviving larvae from treated and control groups were collected and frozen at −80°C for RNA extractions (at least 800 larvae). Total RNA was isolated to analyze *CHS1* expression levels at different *P. citri* developmental stages.

## 5. Conclusions

In conclusion, a full-length cDNA encoding the chitin synthase 1 gene *PcCHS1* was cloned from *P. citri*. The expression profiles of this gene during the *P. citri* developmental stages and under DFB exposure were documented. *PcCHS1* was expressed in all stages, but highly expressed in the egg stage, followed by the larval, nymph, and adult stages. The high expression levels of egg molting might reflect the unique embryo developmental pattern in *Tetranychus. PcCHS1* was up-regulated when the mite was exposed to DFB at LC_30_ and LC_50_. These results indicate that the use of DFB, which may act as an inhibitor of chitin synthesis by up-regulating *PcCHS1*, has promise in *P. citri* control.

## Figures and Tables

**Table 1.** Sequences and relevant information used for phylogenetic analysis of the chitin synthase 1 gene, *CHS1.*

Genes	GenBank No. or Gene ID.	Species
*CHS1*	XP001662200	*Aedes aegypti*
*CHS1*	XP_321336	*Anopheles gambiae*
*CHS1*	XP_395677	*Apis mellifera*
*CHS1*	XP_001866798	*Culex quinquefasciatus*
*CHS1*	NM_079509	*Drosophila melanogaster*
*CHS1*	XP_001359390	*Daphnia pulex*
*CHS1*	ACD10533	*Ectropis oblique Prout*
*CHS1*	AF221067	*Lucilia cuprina*
*CHS1*	GU067731	*Locusta migratoria manilensis*
*CHS1*	JQ040011	*Laodelphax striatella*
*CHS1*	AY062175	*Manduca sexta*
*CHS1*	BAF47974	*Plutella xylostella*
*CHS1*	DQ062153	*Spodoptera exigua*
*CHS1*	AY291476	*Tribolium castaneum*
*CHS1*	tetur03g08510	*Tetranychus urticae*
*CHS1*	KF241748	*Panonychus citri*
*CHS1*	XP003741992	*Metaseiulus occidentalis*
*CHS2*	XP_001651163	*Aedes aegypti*
*CHS2*	XP_321951	*Anopheles gambiae*
*CHS2*	XP_001121152	*Apis mellifera*
*CHS2*	XP_001864594	*Culex quinquefasciatus*
*CHS2*	NM_079485	*Drosophila melanogaster*
*CHS2*	EFX80669	*Daphnia pulex*
*CHS2*	AAX20091	*Manduca sexta*
*CHS2*	ABB97082	*Ostrinia furnacalis*
*CHS2*	XP_002423604	*Pediculus humanus corporis*
*CHS2*	EU622827	*Spodoptera exigua*
*CHS2*	AY525599	*Spodoptera frugiperda*
*CHS2*	AY291477	*Tribolium castaneum*
*CHS2*	tetur08g00170	*Tetranychus urticae*

**Table 2.** Primer sequences used for cloning and quantitative real-time PCR.

Experiments	Primer names and sequences (5′ to 3′)	Product length (bp)
	PcCHS1-1-S: GATGTGACCAAGGGCGTAA	1717
	PcCHS1-1-A: CGCCGAGATTTTTATTTTTC	

Specific PCR	PcCHS1-2-S: CGGAAGCCGTTCAACTATTA	1324
	PcCHS1-2-A: TGTTCCTCGTCTTCCGTCAC	

	PcCHS1-3-S: GTTACCTGGGGAACTCGTGA	1173
	PcCHS1-3-A: TTGTTGAGCGGCTGAAAGTT	

	PcCHS1b-S: TATCTCGCCGATATTGAGGTC	367
	PcCHS1b-A: GATGGAAAAGCATACCGACCA	

3′ RACE	PcCHS1-S1: ATGCTCTTTTCGTTCTCGTG	722

	PcCHS1-S2: TTGTCGGTTTCTTTGGATTA	560

5′ RACE	PcCHS1-A1: TTGGTCACATCCAGCGAAG	503

	PcCHS1-A2: TATCCTGGCAGCACGAA	375

Full-length confirmation	PcCHS1-1: TAACATCCAAATGGGTGCTGA	1000
	PcCHS1-2: GAACCATCAACCAAAAGTCGG	
	PcCHS1-3: CGCTGAATCAATCGGTTTAGGT	1100
	PcCHS1-4: TATCGCTGAAGCAGATGAACGC	
	PcCHS1-5: ATTTACTTTCCATCGGTGCCCA	3131
	PcCHS1-6: CCACGAGAACGAAAAGAGCATT	

*PcCHS1*	PcCHS1-Q-F: AAGTTTGAATACGCGGTTGG	191
	PcCHS1-Q-R: CGATCTTCCCCTTGATCGTA	

*GAPDH*	GAPDH-F: CTTTGGCCAAGGTCATCAAT	159
	GAPDH-R: CGGTAGCGGCAGGTATAATG	
